# Phylogeny, Ecology, and Gene Families Covariation Shaped the Olfactory Subgenome of Rodents

**DOI:** 10.1093/gbe/evad197

**Published:** 2023-11-16

**Authors:** Maxime Courcelle, Pierre-Henri Fabre, Emmanuel J P Douzery

**Affiliations:** Institutdes Sciences de l’Evolution de Montpellier (ISEM), CNRS, IRD, EPHE, Université de Montpellier, Montpellier, France; CIRAD, UMR ASTRE, Montpellier, France; Institutdes Sciences de l’Evolution de Montpellier (ISEM), CNRS, IRD, EPHE, Université de Montpellier, Montpellier, France; Mammal Section, Life Sciences, Vertebrate Division, The Natural History Museum, London, United Kingdom; Institut Universitaire de France (IUF), Section Biologie-Médecine-Santé, Paris, France; Institutdes Sciences de l’Evolution de Montpellier (ISEM), CNRS, IRD, EPHE, Université de Montpellier, Montpellier, France

**Keywords:** olfactory receptor genes, rodents, evolution, pseudogenes, comparative genomics, fossoriality

## Abstract

Olfactory receptor (OR) genes represent the largest multigenic family in mammalian genomes and encode proteins that bind environmental odorant molecules. The OR repertoire is extremely variable among species and is subject to many gene duplications and losses, which have been linked to ecological adaptations in mammals. Although they have been studied on a broad taxonomic scale (i.e., placental), finer sampling has rarely been explored in order to better capture the mechanisms that drove the evolution of the OR repertoire. Among placental mammals, rodents are well-suited for this task, as they exhibit diverse life history traits, and genomic data are available for most major families and a diverse array of lifestyles. In this study, 53 rodent published genomes were mined for their OR subgenomes. We retrieved more than 85,000 functional and pseudogene OR sequences that were subsequently classified into phylogenetic clusters. Copy number variation among rodents is similar to that of other mammals. Using our OR counts along with comparative phylogenetic approaches, we demonstrated that ecological niches such as diet, period of activity, and a fossorial lifestyle strongly impacted the proportion of OR pseudogenes. Within the OR subgenome, phylogenetic inertia was the main factor explaining the relative variations of the 13 OR gene families. However, a striking exception was a convergent 10-fold expansion of the OR family 14 among the phylogenetically divergent subterranean mole-rat lineages belonging to Bathyergidae and Spalacidae families. This study illustrates how the diversity of the OR repertoire has evolved among rodents, both shaped by selective forces stemming from species life history traits and neutral evolution along the rodent phylogeny.

**Significance**

Olfactory receptor (OR) genes represent the largest gene family in mammalian genomes, yet their number strongly differs among species. The causes of the high variability of OR functional and nonfunctional sequences have to be clarified but might be of ecological and/or evolutionary origins. Here, we focused on rodents, the most species-rich mammals, and showed that phylogenetic history and ecological niches such as diet, period of activity, and lifestyle impacted the distribution of OR sequences. Moreover, a convergent expansion of one OR gene family occurred in two phylogenetically independent lineages of subterranean mole-rats. The number and proportion of functional OR and pseudogene sequences detected in rodent genomes have been therefore shaped by their diversified life history traits as well as their evolutionary history.

## Introduction

Among mammals, olfaction is a major chemical sense that played a pivotal role in their evolution and diversification (Aboitiz et al. 2003; Suárez et al. 2012; Rowe and Shepherd 2016). Perception of chemosensory information is notably relevant in mating, feeding, and social behaviors (Doty 1986; Gittleman 1991; Rymer 2020; Smadja et al. 2022). Olfaction involves the binding of odorant molecules from the environment to olfactory receptors (ORs) that are found in the rostral cavity of mammals (Vassar et al. 1993; Buck 2000). One odorant may activate multiple ORs. Conversely, each OR does not have a one-to-one relationship with a single odorant but may bind several molecules. In the brain, activated ORs transmit signals to the olfactory bulb through OR neurons (Buck 1996). The olfactory information of an activated OR is then processed and interpreted by the central nervous system as an odor (Malnic et al. 1999; Saito et al. 2009).

In mammalian genomes, OR genes encode G-protein–coupled receptors (GPCR) and represent the largest multigenic superfamily (Buck and Axel 1991; Niimura and Nei 2007), typically accounting for 4–5% of all coding genes (Hayden et al. 2010). Yet, the number of OR genes is greatly variable among placental mammals: although most taxa have ∼800 to ∼1,200 functional genes, primates typically have about 400 and African elephants have more than 2,000 (Hayden and Teeling 2014; Niimura et al. 2014; Hughes et al. 2018). OR genes encode a family of proteins that display a high variability among-taxon, as any two receptors from a single species may show up to 80% amino-acid sequence divergence (Malnic et al. 2004). Despite such huge variations, OR genes retain a typical structure made up of short (<1 kb), intronless sequences corresponding to seven transmembrane domains displaying conserved aminoacid motifs. These characteristics provide a basis for their in silico extraction and identification from genomic data (Hayden et al. 2014; Han et al. 2022). In addition to these functional genes, mammalian genomes harbor a significant fraction of pseudogenes, which may account for more than 60% of the total OR subgenome (defined as the genomic part comprising OR functional genes and pseudogenes).

In the OR subgenome, the great number and diversity of paralogous sequences led to the identification of different classes of odorant-binding receptor genes. The OR genes are split into class I and class II receptors, which are hypothesized to respectively bind mostly water-borne and air-borne odorant molecules (Freitag et al. 1995). In mammals, class I and II genes represent approximately 10% and 90% of the OR repertoire, respectively (as deduced from Hayden et al. 2010; Hayden and Teeling 2014). Based on genetic sequence similarity, OR genes have been further classified into 17 gene families at a 40% similarity threshold (Glusman et al. 2000; Olender et al. 2004). In model species, analyses at a finer level have enabled the enumeration of 241 OR subfamilies in the mouse and 172 in the human genome with at least 60% similarity in protein sequence (Godfrey et al. 2004; Malnic et al. 2004). Recent phylogenetic analyses demonstrated that the two OR classes can be subdivided into 13 well-supported monophyletic families conserved across mammals: 1) 51, 52, 55, 56 and 2) 1/3/7, 2/13, 4, 5/8/9, 6, 10, 11, 12, 14 (Hayden et al. 2010). To explain the diversity and differences in the number of genes among mammals, it is assumed that the evolution of the OR subgenome follows a “birth and death” model, where new genes arise by duplication of existing sequences, diversify through either neofunctionalization or subfunctionalization, and disappear by pseudogenization (Robertson 1998; Nei and Rooney 2005). Both the number of functional olfactory ORs as well as the ratio of OR pseudogenes versus OR functional genes have often been used as genomic proxies for olfactory ability and acuity in a wide range of vertebrates (Emes et al. 2004; Kishida 2008). This relation is supported by the correlation among 26 mammals between the OR repertoire size and the surface of cribriform plate in the skull (Bird et al. 2018), one of the bony structure that has been used as a morpho-anatomical proxy for olfactory capacities (but see also Van Valkenburg et al. 2011; Martinez et al. 2018, 2020, 2023). A large functional OR repertoire is thought to allow detecting a broader range of odorant compounds, and the pseudogenes provide some clues about ORs turnover with potential gene gains and losses (Nei et al. 2008). Mammals experienced several episodes of such OR subgenome size modifications. African elephants for example underwent numerous duplications and have twice as many OR genes as most sequenced placental mammals (Niimura et al. 2014). By contrast, the number of OR functional and pseudogenes is drastically reduced in cetaceans (Kishida et al. 2007; Hayden et al. 2010).

Several evolutionary forces supposedly drive the OR subgenome dynamics, and their relative contribution has been debated since the discovery of this multigene family (Buck and Axel 1991). Earlier works mainly attributed the variation in the size of OR repertoire between species to ongoing genomic drift (Niimura and Nei 2007; Nei et al. 2008). However, natural selection has also been shown to have a strong impact on the diversity of the OR system. With the increasing availability of genomic data, comparative analyses highlighted the impact of species environment and ecology on OR gene dynamics (Hayden and Teeling 2014). For instance, studies evidenced a consistent loss of OR genes among several aquatic mammals (Kishida et al. 2007; Hayden et al. 2010; Beichman et al. 2019; Liu et al. 2019) or specializations of the OR subgenome toward different dietary niches such as frugivory in bats (Hayden et al. 2014) and various ecological requirements in birds (Khan et al. 2015; Silva et al. 2020). In addition, expansions and contractions of the OR gene repertoire content of extant species may reflect shared phylogenetic constraints. Thus, several studies have specifically taken phylogeny into account when analyzing the relationship between OR genes and adaptation, by using either phylogenetically corrected data or phylogenetic comparative analyses (see, e.g., Hayden et al. 2014; Hughes et al. 2018). However, the extent to which shared ancestry has shaped the OR subgenome similarities in related species is rarely explicitly tested for, even though it has been acknowledged that phylogenetic constraints exist in the morphological structures associated to olfaction (van Valkenburgh et al. 2004; 2014; Macrini 2012; Corfield et al. 2015; Bird et al. 2018; Martinez et al. 2020). Phylogenetic contingency may therefore explain a substantial proportion of the current OR gene diversity as gene gains and gene losses occurring along a given branch of the evolutionary tree may directly affect the descending species. Most of the comparative studies were carried out at a broad evolutionary scale (i.e., placental mammal) with the exception of studies concentrating on Chiroptera (Hayden et al. 2014; Yohe et al. 2022) and indeed focused on OR genes adaptations to ecological variables such as diet or lifestyles, leaving unanswered the question of the importance of phylogenetic inertia concerning the dynamics of the OR genes repertoire (Hayden et al. 2010; Niimura et al. 2014; Hughes et al. 2018; Liu et al. 2019; Yohe et al. 2022).

To better understand the determinants of the dynamics of the OR subgenome evolution in mammals, we focused on Rodentia, the most diversified placental order. Rodents exceed all other mammals in terms of number of species, and they are encountered on each continent but Antarctica. A robust phylogenetic framework is available for rodents, and three major clades comprising mouse, Guinea pig, and squirrel relatives have been evidenced (D’Elía et al. 2019). Moreover, the ecological diversity of this order is striking as they exhibit a wide range of lifestyles and social organization, and they encompass almost every diet recorded in mammals (Single et al. 2001). Rodents adapted multiple times to numerous lifestyles as for the terrestrial, scansorial, or highly arboreal spiny rats of the tribe Echimyini, the subfossorial to highly specialized subterranean mole rats of the families Bathyergidae and Spalacidae, and the semiaquatic beavers and coypus. In addition, an increasing number of genomic assemblies is becoming available for numerous species, opening the possibility to mine and compare large fractions of the OR subgenomes for taxa with contrasted ecological traits and for members of the major evolutionary lineages of rodents (see, e.g., Hargreaves et al. 2017; Lok et al. 2017). For example, the genomes of several fossorial and subterranean rodents have been sequenced, yet the naked mole-rat (*Heterocephalus glaber*) is the only species for which the OR repertoire is fully characterized (Hughes et al. 2018). The subterranean and highly specialized spalacids and bathyergids are known to have convergently lost genes involved in vision (Kim et al. 2011). They developed not only unique modes of communication, such as seismic drummings (Narins et al. 1997; Mason and Narins 2001), but also complex odorant markings (Bennett and Faulkes 2000; Francescoli 2000). Subterranean rodents also use olfactory cues to orient their digging toward food-rich soils (Heth et al. 2002). As such, their olfactory capacities are thought to differ significantly from their terrestrial relatives. Mirroring these physiological features, the expansion of OR family 7 in bathyergids was a proposed marker of their distinctiveness (Stathopoulos et al. 2014). Among other rodents, the OR genes repertoire is available for model species such as the lab mouse (*Mus musculus*) or the brown rat (*Rattus norvegicus*), but limited taxonomic representation in comparative studies overlooks the ecological and phylogenetic diversity in this mammalian order. Numerous studies have pointed out convergent changes and adaptations in the olfactory structures of rodents, making them an excellent model for investigating how phylogenetic and ecological factors influence the development of their OR subgenomes (van Valkenburgh et al. 2011; Martinez et al. 2020).

In this paper, we sought to 1) document the amount of variation in the OR gene repertoire of rodents and 2) understand which evolutionary forces shaped the olfactory repertoire and affected the variation of the number of OR genes among the most diversified mammalian order. To this end, we screened the publicly available genomic sequences of 53 rodent species, encompassing members from almost all major rodent clades. We developed a new method, using similarity-based MCMC and phylogenetic approaches to identify and classify more than 85,000 OR genes, mostly from nonmodel species for which they were not previously described. Improved taxonomic and gene sampling allowed us to incorporate stochastic, phylogenetic, ecological, and traits-related variables to reliably infer and better understand the evolution of the OR repertoire of rodents. Our findings illustrate that the OR repertoire evolution is driven by phylogenetic constraints, ecological and/or physiological tradeoffs, and species life history traits.

## Results

### OR Genes among Rodents

We analyzed 53 rodent genomes spanning the phylogenetic diversity of this taxonomic group and identified and classified 85,355 OR gene sequences into 44,578 mono-exon functional sequences and 40,777 putative pseudogenes (supplementary material S4, Supplemental Material online). Our taxon sampling spans all the major rodent lineages (supplementary table S1, Supplementary Material online) and 80% of their families and also includes two lagomorphs (*Ochotona princeps* and *Oryctolagus cuniculus*) and a primate (*Homo sapiens*) as outgroups. Rodent OR subgenomes had a median of 780 functional genes (64% of the repertoire) and 444 pseudogenes (36%). Class I genes represent on average 9.6% of the functional repertoire, a similar number to what is observed in other mammals (Hayden et al. 2010; Niimura et al. 2014). As our primary data consist of public genomic assemblies obtained through different protocols, we checked that the quality and completeness of assemblies did not impact our results. We did not find any correlation between the total number of genes detected for each species or its fraction of pseudogenes, neither with the number of contigs nor with the contig N50, the sequencing depth, and the Benchmarking Universal Single-Copy Orthologs (BUSCO) completeness score of the assembly (supplementary table S1, Supplementary Material online).

**Fig. 1.**
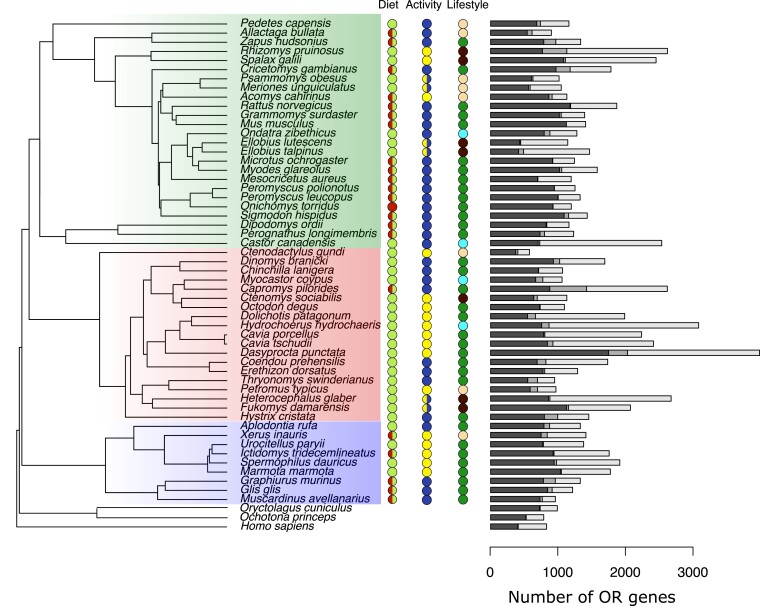
The OR subgenome and ecological traits of 53 rodent species and 3 Euarchontoglire outgroups. Bars represent the number of functional genes (black), full length sequences but bearing less than 7 TM domains (dark gray), and pseudogenes (light gray) identified in this study. Rodent genomes had a median of 780 functional genes (57% of the repertoire) and 444 pseudogenes (43%). The phylogenetic tree is adapted from Upham et al. (2019). Colored areas represent the main rodent clades, that is mouse-related clade (green), Ctenohystrica (red), and squirrel-related clade (blue). For each species, the diet (D), daily activity pattern (A), and lifestyle (L) are depicted: diurnal (yellow)/nocturnal (dark blue)/generalist (half), herbivorous (light green)/insectivorous (red)/omnivorous (half), and desert (light brown)/terrestrial (dark green)/subterranean (maroon)/subaquatic (light blue) taxa.

The number of OR genes varies greatly among rodent species (fig. 1). In our dataset, the Central American agouti (*Dasyprocta punctata*) displayed the largest OR repertoire with 1,737 functional genes and 1,945 pseudogenes. There is more than a 6-fold difference with respect to the species with the smallest repertoire, the common gundi (*Ctenodactylus gundi*), which only had 370 functional and 202 pseudogenes. Biological model species such as the house mouse (*M. musculus*) and brown rat (*R. norvegicus*) are among the species with the highest number of functional genes with 1,116 and 1,170 functional copies respectively.

When OR gene numbers were mapped onto a well-accepted phylogeny of rodents, the Pagel's lambda parameter was estimated at 1, which denotes a strong phylogenetic signal in the data analyzed. In other words, the number of OR genes mostly varied according to a Brownian motion (BM), that is the degree of resemblance among the OR subgenomes of rodents diverged according to a random walk along the chronogram. Taking these results into account, we found a significant link between the number of OR pseudogenes and the number of functional ORs (*P* value = 0.0026). However, there was no link between the fraction of pseudogenes and the number of functional genes (*P* value = 0.33).

### Ecological Traits Impact the OR subgenome

**Fig. 2.**
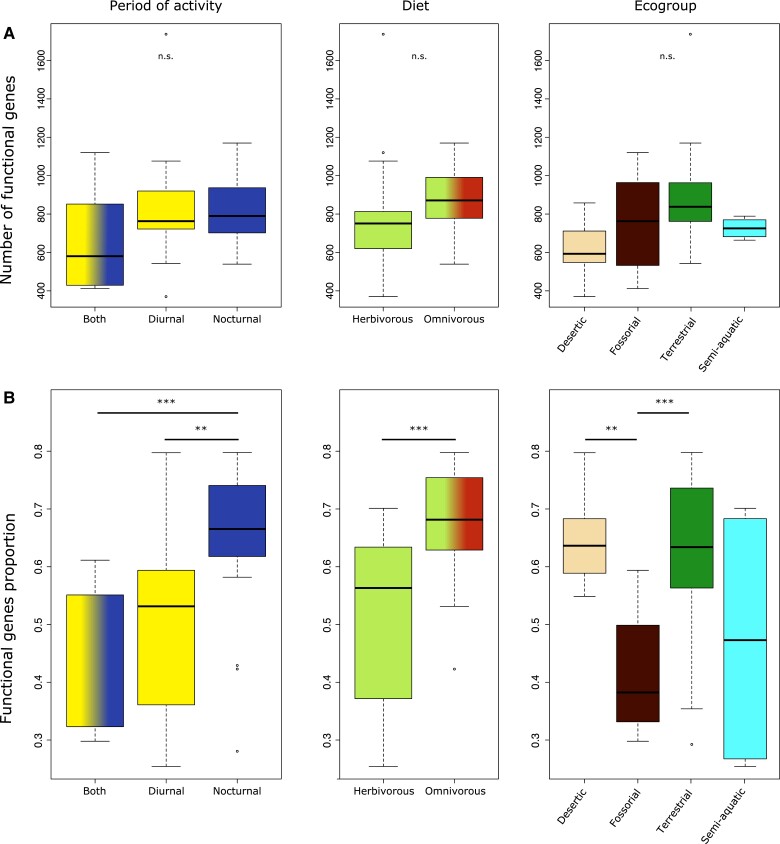
Number of functional OR genes (*A*) and fraction of functional genes (with respect to the total number of functional genes and pseudogenes) in the OR subgenome (*B*) for each ecological niche in 53 rodents. The results of pairwise phylogenetic Tukey comparisons are reported (n.s., not significant; *P value < 0.05; **P value < 0.01; ***P value < 0.005). Results are not displayed on (*A*) as no comparison was significant.

To investigate whether ecological traits potentially influenced the OR rodent subgenome, we compared the number of functional OR genes and the fraction of pseudogenes of species according to each factor of their activity period, diet, and lifestyle (fig. 2). To correct for the species similarity due to common ancestry, Tukey's Honestly Significant Difference (HSD) tests were performed assuming a BM of the number and fraction of functional genes along the phylogeny (supplementary table S5, Supplementary Material online). Diurnal and nocturnal rodent species have more functional OR genes than generalist ones, here defined as species not specialized toward a specific diurnal or nocturnal activity (median, respectively, 763 and 790 vs. 580 genes), although the difference is not statistically significant (fig. 2*A*). However, nocturnal species exhibited a significantly smaller proportion of pseudogenes than both diurnal (Tukey's HSD *P* value: 5.9e−3) and generalist (*P* value = 3.7e−4) ones (fig. 2*B*).

We then evaluated the diet impact on the OR subgenome content. Omnivorous and insectivorous species tended to have more genes than herbivorous rodents, although the difference was not significant. Contrastingly, the proportion of pseudogenes was significantly lower than in herbivorous species (median 0.68 vs. 0.56, respectively, *P* value = 1e−5). Finally, there was no significant effect of ecological preferences on the absolute number of functional genes. For fossorial species, we however detected a significantly higher frequency of pseudogenes (median 63.9%) when compared with all terrestrial and desertic species (median 36.7% and 37.1, respectively). Among semiaquatic species, the North American beaver (*Castor canadensis*) and capybara (*Hydrochoerus hydrochaeris*) display an unusually large fraction of pseudogenes (72.0% and 74.6%, respectively), but the muskrat (*Ondatra zibethicus*) does not.

### Evolutionary Processes Influencing the Composition of the OR Genes Repertoire

**Fig. 3.**
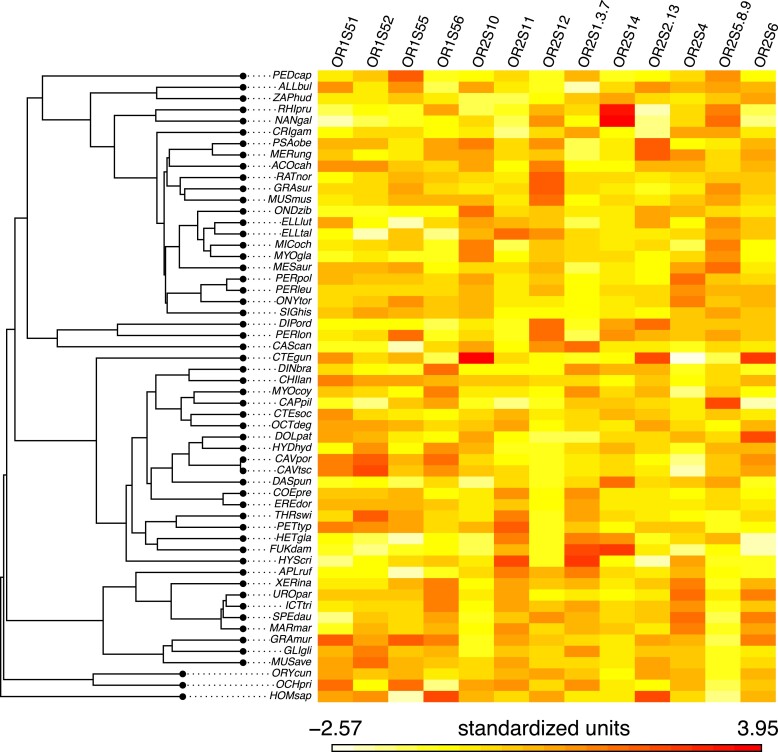
Phylogenetic heatmap of the 13 OR gene families proportions in the OR subgenome. OR functional genes number in each family (from OR1S51 to OR2S6) was divided by the total functional gene number for that species. Each column was then standardized, so that each family has a mean of 1. The phylogenetic tree is adapted from Upham et al. (2019). Full species names are reported in supplementary table S1, Supplementary Material online.

To better characterize the evolution of the OR gene repertoire of rodents, we examined the subgenome dynamics at the levels of its two gene classes and its 13 gene families. To remove the effect of the among-species variability in the absolute number of genes, subsequent analyses were conducted on the proportion each family takes in a given species functional OR subgenome (fig. 3).

**Fig. 4.**
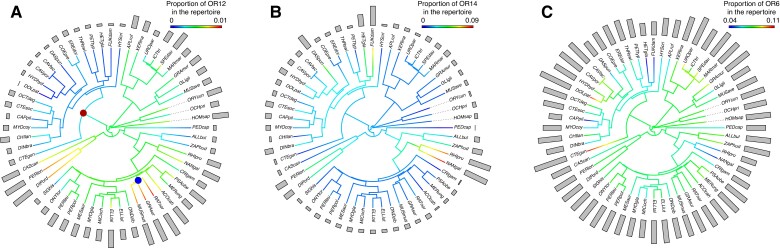
Maximum-likelihood reconstruction of ancestral proportion of three example OR gene families. Current species' proportions were mapped to the rodent phylogenetic tree of Upham et al. (2019). Ancestral states were then estimated using the « fastANC » function of the *Phytools* R package (Revell 2012). In the small family 12 (*A*), the ancestor of hystricomorph species (node marked red) likely lost several genes, whereas for murids species (node marked blue), the number of genes increased. Gene family 14 (*B*) displays an important expansion in the fossorial lineage Bathyergidae and Spalacidae (on average 7.3% of all functional OR, vs. 1.5% in other rodents). In contrast, these species show a reduction of the family 6 (*C*) (4.5% of the OR repertoire vs. 7.5% in other rodents). Full species names are reported in supplementary table S1, Supplementary Material online.

Using Pagel's lambda tests, we found that 11 of the 13 OR families were significantly affected by phylogenetic inertia. Evolutionary history therefore had a significant impact on the OR genes repertoire composition of extant rodent species. Families 52, 56, 1-3-7, 10, 11, 14, 2-13, and 5-8-9 displayed a strong phylogenetic signal (λ > 0.90). Families 51, 4, and 12 showed a weaker but still significant signal (λ equals 0.73, 0.64, and 0.79, respectively). For families 55 and 6, proportions of genes were not statistically distinguishable from a random distribution given our phylogeny (likelihood ratio test *P* value = 1 in both cases). An example of phylogenetic constraints can be seen on the OR gene family 12 (fig. 4*A*). In this small family (<1% of the total repertoire), the last common ancestor of Ctenohystrica (guinea pig-related rodents, fig. 4*A* red node) likely lost several OR12 genes, leading to its modern descendants having fewer genes (median: 1) than squirrel-related (4 genes) and mouse-related species (5 genes). Conversely, murids rodents (fig. 4*A* blue node) experienced more duplications in this gene family (median 11 genes).

**Fig. 5.**
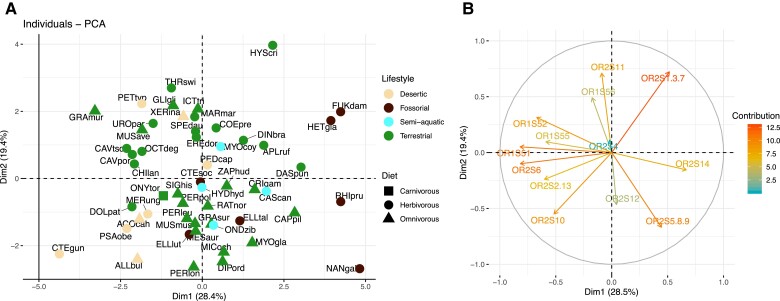
PCA of the proportion of OR gene families (*A*) and corresponding circle of correlations of OR families to PC 1 and 2 (*B*). The first two PCs account for 47.8% of the total variance. OR families 51, 6, 52, and 55 load negatively to PC1, whereas families 14 and 2-13 load positively to this PC. Families 1-3-7, 11, and 56 contribute positively to the second component, and families 5-8-9, 10, and 12 load negatively to PC2. Symbol color represents species ecogroups (beige: desertic, brown: fossorial, blue: semiaquatic, green: terrestrial). Symbol shape depicts diet (square: carnivorous, disc: herbivorous, triangle: omnivorous). Full species names are reported in supplementary table S1, Supplementary Material online.

**Fig. 6.**
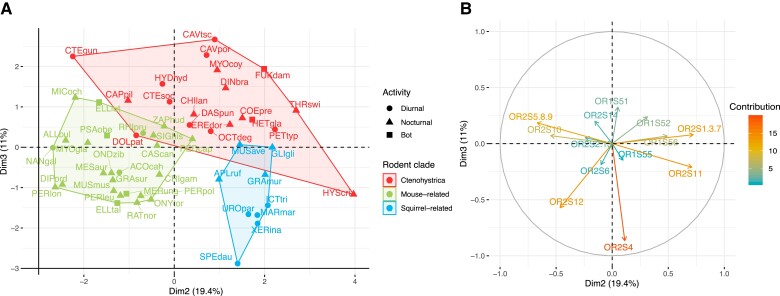
Second and third PC of the PCA on the proportion of OR gene families (*A*) and corresponding circle of correlations of OR families to PC 2 and 3 (*B*). Second and third PC account for 30.4% of the total variance. Symbol color and polygons represent rodent’s major clades (red: Ctenohystrica, green: mouse-related species, blue: squirrel-related species). Symbol shape depicts the daily activity patterns (disc: diurnal, triangle: nocturnal, square: both). Full species names are reported in supplementary table S1, Supplementary Material online.

The model that best fits the relative importance of the different gene families in the OR subgenome is an OU model with only one adaptive optimum corresponding to the lifestyle categories (supplementary table S6, Supplementary Material online). Thus, in contrast to the overall proportion of functional OR genes, the diet and activity period do not seem to have a consistent effect on the content of gene families. To visualize this trend, we performed a principal component analysis (PCA) on the normalized proportion of functional genes in each family per species (see Methods, figs. 5 and 6). The first two principal components (PCs), respectively, account for 28.4% and 19.4% of the total variance. OR families 51, 6, 52, 2-13, and 55 strongly load negatively to PC1 (contribution of 18.1%, 18.0%, 12.1%, 9.8%, and 9.0% respectively), whereas family 14 loads positively to this PC (12.0% contribution). Families 1-3-7, 11, and 56 contribute positively to the second component (contribution of 21.3%, 20.6%, and 9.8%), and families 5-8-9, 10, and 12 load negatively to PC2 (contribution of 18.3%, 12.3%, and 8.8%). As shown in the individual plot (fig. 5*A*), four species have unusually high values on the PC1: *H. glaber*, *Fukomys damarensis*, *Rhizomys pruinosus*, and *Spalax galili*. These species belong to the Bathyergidae and Spalacidae families, both characterized by their subterranean lifestyles. They exhibit a low relative proportion of class 1 and OR6 gene families as well as a notable expansion of family 14, which makes up to 9% of the total OR subgenome (median 1.3% in other species). The OR subgenome of the other subterranean fossorial lineages available in our dataset—the genera *Ellobius* and *Ctenomys*, respectively, belonging to Cricetidae and Ctenomyidae families—do not display these features. Two other species display outlier positions on the two first PCs: the common gundi (*C. gundi*) shows low values on both PC1 and PC2, as it exhibits unusually high proportions of families 10 and 2-13, and 6 in its OR repertoire. Conversely, the crested porcupine *Hystrix cristata* contributes to ∼12.5% of PC2 alone, as its families 11 and 1-3-7 are about twice as large as in other rodent lineages here sampled.

When we consider the second and third PCs (19.4% and 11% of total variance), there is a clear phylogenetic pattern (fig. 6) with species groups corresponding to the mouse-related, squirrel-related, and Ctenohystrica clades. Specific characteristics of mouse-related species OR subgenome thus include a higher proportion of genes from families 5-8-9 and 12 and lower number of genes from families 11 and 1-3-7 than squirrel-related species. Members of Ctenohystrica have on average a lower proportion of family 4 genes (11% of the total OR repertoire on average vs. 14% for other rodents) but occupy more space on the PC2, meaning their composition in genes of families associated to this PC (1-3-7, 11, 56, 5-8-9, 10, and 12) is more diverse than in other clades.

## Discussion

### Variability in the OR Gene Numbers

In this study, we retrieved a total of approximately 85,000 OR functional and nonfunctional genes from the genomes of 53 rodent species. It is important to note here that we use the word “functional” mainly in the bioinformatic sense of the term, that is generally as opposed to fragmentary sequences or sequences inactivated by the presence of stop codons. However, we have taken out an extra step here by removing genes that do not have the seven transmembrane domains characteristic of functional GPCRs. This analysis allowed us to correctly label 4,049 sequences of the correct length but with fewer than 7 transmembrane helices. This step allowed us to refine our results, although this is still a relatively simple model of protein functionality and will not replace controlled activation assays.

The per-species OR gene numbers are close to previous analyses of rodent genomes, with a maximum 1% to ∼4% difference with respect to taxa shared with Hughes et al. (2018). These minor discrepancies are likely due to differences in OR recovery methods and genomic assembly datasets. As in other mammals (Niimura et al. 2014, Hughes et al. 2018), the number of OR genes is contrasted among rodents. Rodents total OR repertoire size variability is similar to the one displayed by the rest of nonaquatic mammals, ranging from 572 (*C. gundi*) to 3,960 (*D. punctata*) functional OR + pseudogenes in our dataset. Compared with other mammals, *D. punctata* ranks among the largest known OR repertoire sizes, behind the African elephant *Loxodonta africana* (Niimura et al. 2014; Hughes et al. 2018). Although the number of genes in rodents is on average slightly higher than in other mammals (1,524 on average compared with 1,213), some species have relatively few. Several taxa such as *C. gundi* (Ctenodactylidae), *Allactaga bullata* (Dipodidae), or *Thryonomys swinderianus* (Thryonomyidae) have a number of OR genes comparable with primates. The same conclusions also hold for the proportion of functional genes in the OR repertoire, which ranges from ∼76% (*Sigmodon hispidus*) to 25% (*H. hydrochaeris*). According to previous analyses (Niimura et al. 2014), the number of functional OR genes did correlate with the number of pseudogenes. On the contrary, the fraction of OR pseudogene cannot be linked to the number of functional genes among rodents: species with similar fraction of pseudogenes such as *C. gundi* and *Myodes glareolus* (0.32 and 0.34, respectively) show almost a 3-fold difference in term of functional genes number (370 and 1,014, respectively). Thus, the fraction of OR pseudogenes should not be used as a proxy of the olfactory capacities of rodent species.

### Links between Olfaction and Ecological Traits

The variation in the number of genes and fraction of pseudogenes in this study suggests that ecological traits play an important role in the evolution of the OR subgenome. Studies in other vertebrates have reported that nocturnal species have a larger main olfactory bulb than diurnal species, which has been linked to a more important use of olfactory cues in species which forage at night when vision is limited (Barton et al. 1995; Barton 2006; Steiger et al. 2009). Among rodents, nocturnal species do not appear to share a consistently higher number of functional genes than their diurnal counterparts. In contrast, nocturnal taxa in our sample retain a higher proportion of functional genes when compared with diurnal or generalist species (fig. 2). The substantial difference in the number of OR among rodents may explain why we do not detect a clear effect of ecological traits on the raw number of genes. Indeed, among all chemosensory receptor types, the number of ORs has been shown to have the highest variance and the lowest statistical association with diurnality, even in a relatively small sample of taxa at the mammalian scale (Wang et al. 2010). Consequently, a better way to analyze this association may be to sample pairs of related species with different ecologies and test whether ecological traits have a convergent effect along the phylogeny. Indeed, from the four ecogroups sampled here—terrestrial, desert, fossorial, and subaquatic—fossorial species stand out (fig. 5). This lifestyle affected both the overall gene family composition and the fraction of pseudogenes of the OR subgenome. Four fossorial rodents display reduced proportions of class I and OR gene family 6 as well as an expansion of OR gene family 14 (figs. 4*B* and *C* and 5). These species belong to two families, which independently adapted to the subterranean lifestyle, namely Spalacidae (*R. pruinosus* and *S. galili*) and Bathyergidae (*F. damarensis* and *H. glaber*). Of note, our analyses did not recover an expansion of the OR gene family 7, which was otherwise proposed as another genomic adaptation to fossoriality (Stathopoulos et al. 2014). Family 7 was not studied on its own here but grouped with families 1 and 3, with which it forms a monophyletic gene cluster (Hayden et al. 2010). Bathyergids do exhibit a high proportion of family 1-3-7, but it is not shared by other fossorial lineages (figs. 3 and 4). Moreover, some nonfossorial rodents show a similar or higher proportion of 1-3-7 genes compared with bathyergids. As the dataset from Stathopoulos et al. (2014) did not include other Ctenohystrica rodents than *Cavia porcellus,* nor other fossorial lineages, these authors did not have the possibility to evaluate whether or not the expansion of family 1-3-7 might be a clade-specific trait of bathyergids instead of an adaptation to an ecological niche. However, Stathopoulos et al. (2014) detected positive selection on the binding domains of OR from the gene family 7, which may highlight a functional role to this gene expansion. Fossorial rodents indeed developed a wide set of such sensory adaptations to their underground lifestyle, which include a degraded vision and hearing (Heffner and Heffner 1993), whereas experimental tests have demonstrated a high olfactory acuity in several species (Judd and Sherman 1996; Lange et al. 2005; Onyono et al. 2017). Here, Spalacidae and Bathyergidae have convergently developed a unique subgenomic signature with this 10-fold OR family 14 expansion, which is not shared by other fossorial lineages such as the subterranean genera *Ellobius* or *Ctenomys*. Although these species all share morphological adaptations to digging and spend most of their lives in burrows, they represent varying degrees of fossoriality. *Ctenomys* species regularly exit their burrows at night to feed above ground (Begall et al. 2007; Šumbera 2019). As such, it would make sense that the olfactory capacities of these animals remain close to those of their surface-dwelling parents. Bathyergidae and Spalacidae are ancient lineages that likely adopted a subterranean lifestyle 31–24 Ma (Patterson and Upham 2014; Steppan and Schenk 2017), in contrast to *Ellobius*, an Arvicolinae genus which diversified only 5 Ma (Steppan and Schenk 2017). Other senses such as vision were reported to not be as modified in *Ellobius* as in more strictly subterranean rodents (Herbin et al. 1994). Considering the extent of phylogenetic inertia highlighted in the OR repertoire, we can assume that the composition of the OR subgenome in *Ellobius* has not been impacted by underground life as strongly as those of Bathyergidae and Spalacidae. The nature of this OR repertoire adaptations is otherwise still unclear. The OR family 14 may encode receptors particularly well-suited for underground-living rodents, and positive selection could thus explain the 10- fold expansion observed in Bathyergidae and Spalacidae. In this hypothesis, family 14 OR genes would either bind ligands associated to underground food resources or be more efficient for underground environments. Among mammals, such adaptations of the OR repertoire to diet (Luca et al. 2010; Hayden et al. 2014) or lifestyle (Hayden et al. 2010) have been evidenced. However, ligands of family 14 receptors are still mostly unknown as genes of this family have not been de-orphanized. Thus, we were not able to pinpoint specific OR genes or ligands especially adapted to underground dwelling in our dataset. In the absence of a more functional characterization of these OR genes, other nonolfactory processes might also provide insights into the subterranean adaptations of Bathyergidae and Spalacidae. Underground life poses major stresses for rodent species: a hypoxic and hypercapnic environment and high temperature and humidity (Zelová et al. 2007). Peculiar features once thought to characterize *H. glaber*, such as unusual longevity and cancer resistance, have since been expanded to other bathyergids such as *Fukomys* (Dammann et al. 2011) and even convergently evolved in *Spalax* species (Gorbunova et al. 2012; Manov et al. 2013). As it is now established that OR genes may be expressed outside of the olfactory epithelium and some of them were even shown to either promote or inhibit tumor formation (Sanz et al. 2014; 2016; Kalbe et al. 2017), OR genes may even prove to be interesting candidates to metabolic adaptations of the underground lifestyle.

Another ecological change with major implications for the sense of olfaction is the shift to a semiaquatic lifestyle from a terrestrial ancestor. This phenomenon is well described in the literature and convergently leads to pervasive reductions of the OR subgenome as well as increased pseudogenization among several clades of aquatic mammals (Kishida et al. 2007; Beichman et al. 2019; Huelsmann et al. 2019; Liu et al. 2019) and also aquatic snakes (Kishida et al. 2019). This genomic footprint is also accompanied by morphological clues, as the olfactory capabilities and morphological structures related to olfaction in small amphibious mammals are typically reduced due to thermoregulation constraints (Martinez et al. 2020). Among the four lineages which independently acquired a semiaquatic lifestyle in our sampling, the beaver (*C. canadensis*) and capybara (*H. hydrochaeris*) partially follow this trend. Although their functional gene number is similar to that of their terrestrial close relatives (*Perognathus longimembris* and *Dolichotis patagonum*, respectively), they convergently exhibit the two highest fractions of pseudogenes of our sampling, 72% and 75%, respectively (fig. 1). This unusually high number of inactivated OR sequences might reflect the relaxation of selective constraints on the OR subgenome following the shift of the beaver and capybara to a more aquatic environment. Contrastingly, the coypu and muskrat did not display convergent evolution, neither in the number of OR genes nor in the fraction of pseudogenes (fig. 1) nor in the OR gene family content (fig. 4). Regarding the retention of both olfactory OR genes (results herein) and olfactory bony structures (Martinez et al. 2020) in amphibious species, it has been documented that semiaquatic rodents may still rely on terrestrial olfactory-based scents for foraging (Müller-Schwarze et al. 1994), mating (Macdonald et al. 2007), and territorial behaviors (Rosell et al. 1998). In this regard, it makes sense that the OR repertoire of the coypu and muskrat is still under selection and did not degenerate as much as the OR subgenomes of fully secondary-adapted aquatic mammals such as cetaceans or manatees. In future studies, the OR repertoire of other semiaquatic lineages such as *Crossomys moncktoni*, the “most highly specialized amphibious murid” (Helgen 2005), would provide other examples of terrestrial to amphibious ecological shifts to further investigate the conflicting patterns we have evidenced. Other investigation avenues would include transcriptomics approaches on olfactory anatomical structures to assess the degree of variability in the OR gene expression levels between tissues and taxa (Yohe et al. 2022). A study of OR dynamics, using pseudogenes as well as functional genes, would also provide information on gene loss and gain along specific branches of the phylogeny, which in turn would allow more specific investigations of OR number variation during major ecological shifts.

### Gene Families Composition of the OR Subgenome

In order to have more resolution on the evolution of the OR repertoire, we broke down OR genes into 13 OR gene families as defined in Hayden et al. (2010), which are hypothesized “gene clades.” However, as very few nonsynonymous substitutions can change the binding affinities to the ligand (Gaillard et al. 2004; Katada et al. 2005), there is no guarantee for phylogenetically related genes to be functionally similar. Still, as the detailed mechanisms of ligand binding are unclear and relatively few OR have been deorphanized, they provide a phylogenetic framework in the absence of more functional categorizations (e.g., Hayden et al. 2014; Hughes et al. 2018). As we focus here on the expansion or reduction of the different gene families, we normalized the number of genes in each family by the total number of genes for each species, so that families are represented by the proportion of the OR repertoire they account for. Using this transformation, we were able to detect a strong phylogenetic signal on 8 of the 13 OR families and a weaker but still significant signal in 3 of the 5 remaining families. Similarities in the proportions of the classes in the OR subgenome of different species are thus largely explained by their shared common ancestry. Although it has been demonstrated that phylogenetic inertia constrains the evolution of the size of the olfactory bulb size (Corfield et al. 2015), this is the first time to our knowledge that this hypothesis is tested on the OR gene repertoire. As opposed to the ratio of functional genes over pseudogenes, the diet and period of activity of species did not strongly influence the proportion of the different OR families in the repertoire. Only in the fossorial taxa did we detect a strong signal for an expansion of family 14 and a reduction of class I families. Rather, the composition of the OR subgenome accurately reflects the phylogenetic relationships among rodent species (fig. 6). This property might indicate that niche adaptation did not lead to gene family-specific losses and duplications but instead operated at the OR subgenome level. The proportion of OR families would then have evolved neutrally during the rodent history, leading closely related species to display a similar subgenome composition regardless of their life history traits. Thus, the phylogenetic position of a species seems to be a reasonable predictor of the relative importance of the different gene families in the OR subgenome.

### Conclusion and Future Directions

In summary, we used 53 publicly available genomes to analyze the structure of the OR genes repertoire for the major clades of the order Rodentia. We showed that different ecological and evolutionary forces act on different facets of the rodents OR subgenome. In our dataset, diet, period of activity as well as lifestyles of rodents potentially contributed to shape their number of functional OR genes and the proportion of pseudogenes. However, both the number and the proportion of 13 gene OR families seem to be related to the divergence of the major rodent clade. A notable exception was the convergent 10-fold expansion of the OR family 14 in two phylogenetically distant subterranean clades, the Bathyergidae and Spalacidae families. This study highlights precautions to be taken when extrapolating evolutionary processes over clades, as clade-specific patterns may blur the evolutionary signal. An effective method to address this problem would be to study the pairs of closely related species to test for convergence. This design would also prevent autapomorphic patterns to be interpreted as clade-specific or ecology-specific traits. Although our taxonomic sampling strongly improves our knowledge about the OR subgenome structure among rodents, future directions for research also include investigation of the functional relevance of the gene family classification when studying the relation between olfaction and ecology, as well as the evolution and the dynamics of OR pseudogenes.

## Material and Methods

### Genomic Datasets

Genomic assemblies of 53 species belonging to the major rodent clades were downloaded from publicly available databases. For each assembly, we have retrieved the number of contigs, the contig N50, and the sequencing depth when available. Genome completeness was further evaluated by BUSCO v4.0.5 (Seppey et al. 2019) with the “glires” database. These statistics as well as the accession number of genomic assemblies are summed up in supplementary table S1, Supplementary Material online.

### Pipeline of OR Genes Detection

In order to extract OR genes from mammalian genomes and to assign them to their respective OR families, we developed an in-house flexible pipeline. OR sequences from *H. sapiens*, *Canis lupus*, *M. musculus*, *R. norvegicus*, and *Ictidomys tridecemlineatus* were downloaded from Ensembl and distributed among monophyletic families according to Hayden et al. (2014). These sequences were aligned using MAFFT v7.271 and then used as a starting point to build an HMM database per OR family using the hmmbuild program of the HMMER 3.2.1 suite (Eddy 2011).

Rodent OR gene sequences were mined from genomic assemblies with the nhmmer program. HMM hits from every OR family were then brought together, and we used the CD-HIT v4.6.8 (Fu et al. 2012) software to merge redundant sequences and ensure that each individual OR gene was present only once. We used a similarity threshold of 98% to account for different alleles of the same gene, sequencing, and assembly errors and to be consistent with existing literature using this threshold (e.g., Hughes et al. 2018). To remove spurious sequences that may belong to close but non-OR GPCR protein families, we applied a phylogenetic filter to the HMM result sequences. Each putative sequence was aligned against 26 verified *M. musculus* OR sequences (2 from each OR gene subfamily, extracted from the Ensembl database) and 11 non-OR GPCR genes following Niimura (2013). Phylogenies were then inferred for each new individual sequence using FastTree (Price et al. 2010) under an approximation of the CAT-GTR-Gamma evolution model. Finally, sequences that clustered with the non-OR outgroup were removed from the dataset. This approach sets our pipeline apart from other methodologies. This procedure allowed us Hothorn et al. (2008) to detect and eliminate several non-OR gene sequences that yet passed HMM-based tests on which other available pipelines are currently based (Hayden et al. 2010, Han et al. 2022). Such outlier sequences belonged to other GPCR gene families, namely melanocortin-1 receptors, trace amine-associated receptor 2, and 5-hydroxytryptamine receptor 1A (supplementary fig. S2, Supplementary Material online).

Sequences that contained at least one stop codon and/or one frameshift mutation were tagged as “pseudogenes.” As the minimum required length to code for seven transmembrane domains is 650 bp, shorter sequences were also classified as nonfunctional. Remaining sequences were labeled as “functional.” We then used the perl module ORA (Hayden et al. 2010) to assign each gene to an OR family.

This model implies that in a given genome assembly, a functional sequence truncated to a length inferior to 650 bp would also be categorized as a pseudogene. We acknowledge that this could have led to overestimate the number of pseudogenes, but this strategy nevertheless provides a conservative estimation of the functional OR gene number. This issue should be more prevalent in the most fragmented assemblies as there is a greater chance that a functional sequence will be truncated. To assess if this issue occurred in our datasets, we measured the correlation between the fraction of pseudogenes detected for each species and the number of contigs, the contig N50, sequencing depth, and BUSCO completeness score of the assembly using the cor.test function in R (R Core Team 2017).

Our pipeline is freely available from github (https://github.com/CourcelleM/OR_Finder/). The associated scripts are encapsulated in a Snakemake workflow (Köster and Rahmann 2012), which makes it easy to install, run, and scale on any hardware or computing cluster. A summary of the main pipeline steps is also provided (supplementary fig. S3, Supplementary Material online).

### Assessing Functional Sequences

We used the deepTMHMM software (Hallgren et al. 2022) to identify the characteristic seven trans-membrane (TM) helices domains of OR genes in the aminoacid translation of sequences judged by the pipeline to be “functional.” Nine percent of these sequences were found to have six or fewer TM domains, highlighting that filters based on sequence length alone may not be sufficient to discriminate true “functional” gene sequences. Only sequences with seven TM domains were flagged as functional for further analyses.

### OR Genes Phylogeny Inference

In order to assess the relevance of the gene family assignment, we clustered all our functional genes dataset independently from the family clustering sensu Hayden et al. 2010. All functional sequences were first aligned at the aminoacid level with MAFFT (Nakamura et al. 2018) and then back at the nucleotide level with the “reportGapsAA2NT” program of the MACSE software (Ranwez et al. 2011). Sites with more than 80% missing data were removed from the alignment using trimAl v1.4 (Capella-Gutiérrez et al. 2009). An approximately maximum-likelihood phylogenetic tree was computed using a GTR model of nucleotide evolution and 20 different site evolution rates in FastTree (Price et al. 2010).

### Ecological Traits Impact on the OR Subgenome

We used the “fitContinuous” function of the geiger (Pennell et al. 2014) package in R to fit BM and Ornstein–Uhlenbeck (OU) models to the number of functional genes. The best fitting model was determined using the Akaike's Information Criterion (AIC), which weights the likelihood of the model against its number of parameters. Here, the BM and OU models had a similar AIC (−64.3 and −65, respectively), so we used the simpler BM model for the following tests. Multiple pairwise Tukey comparisons were then performed with the “glht” function of the multcomp (Hothorn et al. 2008) package to test for differences among our samples taking the BM models into account. The same methodology was applied to the fraction of functional pseudogenes.

### OR Family Composition, Ecology, and Phylogenetic Signal

Due to variations in the number of OR, it is challenging to study intergene families variation between species. To remove the effect of the absolute number of genes, we conducted analyses on the proportion of each family in a given species functional OR repertoire. We first tested the strength of phylogenetic constraints using Pagel's lambda tests (Pagel 1999), implemented in the “phylosig” function of the phytools R package (Revell 2012). The lambda statistic ranges from 0 when traits evolve independently from the phylogeny to 1 when the trait follows a BM model. Likelihood ratio tests were used to test whether lambda significantly differs from 0. The effect of species diet, activity period, and lifestyle on the family composition of genes in their OR subgenome was then assessed. We recorded from the literature diet, activity period (diurnal vs. nocturnal), and lifestyle (see supplementary table S1, Supplementary Material online). Using stomach content data, we placed each species into three distinct categories omnivoran (species that include both animal and plant items), herbivoran (species that include only or mostly plant items), and animalivorans (species that include only or mostly animal items). In our lifestyle category, we considered species with extreme adaptation toward fossoriality (subterranean rodents according to Lacey 2000), desertic life (species known to have multiple desert morphological and behavioral adaptations and known to mostly occur in desert habitats), and amphibious lifestyle. First, we used the mvgls function from the mvMORPH package (Clavel et al. 2019) to fit linear models to the proportion of genes in each OR family, normalized so that each family had the same weight regardless of its absolute number of genes. We fitted one BM model, three OU models with lifestyle, diet, and activity period as explanatory variables, and one OU model with all three ecological variables combined. The best fitting model was determined using the generalized information criterion (Clavel and Morlon 2020). A Multivariate ANOVA (manova.gls function from mvMORPH) was then used to assess model fit and significance of variable effects (Clavel and Morlon 2020). To visualize the proximity of species according to the composition of their OR subgenome, we ran a PCA using the “dudi.pca” function of the ade4 package. As for the model-fitting, PCA input was the normalized proportion of genes in each OR family.

## Supplementary Material

evad197_Supplementary_Data

## Data Availability

The data underlying this article are available in the article and in its online supplementary material (see supplementary table S1, Supplementary Material online for the genome accessions).
