# Supplementary material for: Phylogeny, Ecology, and Gene Families Covariation Shaped the Olfactory Subgenome of Rodents
Source: Genome Biol Evol. 2023 Nov 16;15(11):evad197. doi: 10.1093/gbe/evad197 (PMC10653590; doi:10.1093/gbe/evad197)
Supplement: evad197_Supplementary_Data [file evad197_supplementary_data.zip › SUPPLEMENTARY TABLE AND FIGURE CAPTIONS.docx]

**SUPPLEMENTARY TABLE AND FIGURE CAPTIONS**

**Table S1**. Genomic dataset used in this study in csv format. The species sampled, genome accession numbers, assembly statistics, ecological traits and number of functional and pseudogene sequences are reported.

**Fig. S2**. Maximum likelihood phylogenetic tree inferred from all olfactory receptors sequences annotated in this analysis, before adding a phylogenetic filter to our annotation pipeline. The different colors represent sequences from the 13 OR gene families. Several non-OR sequences can also be detected, belonging to three other protein groups: MC1R, TAAR2 and HTR1A. The sequences are removed when using a phylogenetic control, *i.e.* putative sequences must group with known OR genes against other GPCR proteins (See Fig. S3 and Materials and Methods).

**Fig. S3**. OR annotation flowchart, adapted from Niimura (2013) to summarize our pipeline process. The main steps of the pipeline are described. 1) Sequences are extracted from genomic contigs using an HMMER search (Eddy 2011) against a database of known OR sequences from several mammal species. 2) For each putative OR sequence, we then apply a phylogenetic filter by building a tree from the putative sequence, known OR genes and several related, non-OR GPCR proteins as suggested by Niimura (2013). Sequences that do not group with OR genes are removed from the dataset as they likely belong to another gene family. 3) Sequences are then labeled functional if their coding sequence is strictly longer than 650 nucleotides, the minimum required length to code for seven transmembrane domains (Hayden et al. 2014). Otherwise, sequences are labeled as pseudogenes. 4) Finally, we use the ORA perl script (Hayden et al. 2014) to assign the sequences to the different OR gene families.

Supplemental Material S4. All functional olfactory receptor sequences detected in this study in fasta format.

Table S5. Table summary of Tukey’s HSD results. We compared the effect of rodents’ species period of activity, diet and ecogroups on the number of functional OR genes, and the proportion of functional in the species OR repertoire.

Table S6. Table summary of model fitting to the proportion of OR gene families in the OR repertoire. Four models were contrasted: one brownian movement (BM) model, three Ornstein-Uhlenbeck (OU) models with either lifestyle, diet and activity period as explanatory variables, and one OU model with all three ecological variables combined. Model fitting was determined using the Generalized Information Criterion (GIC).
