# Supplementary figures and images for: Phylogeny, Ecology, and Gene Families Covariation Shaped the Olfactory Subgenome of Rodents
Source: Genome Biol Evol. 2023 Nov 16;15(11):evad197. doi: 10.1093/gbe/evad197 (PMC10653590; doi:10.1093/gbe/evad197)

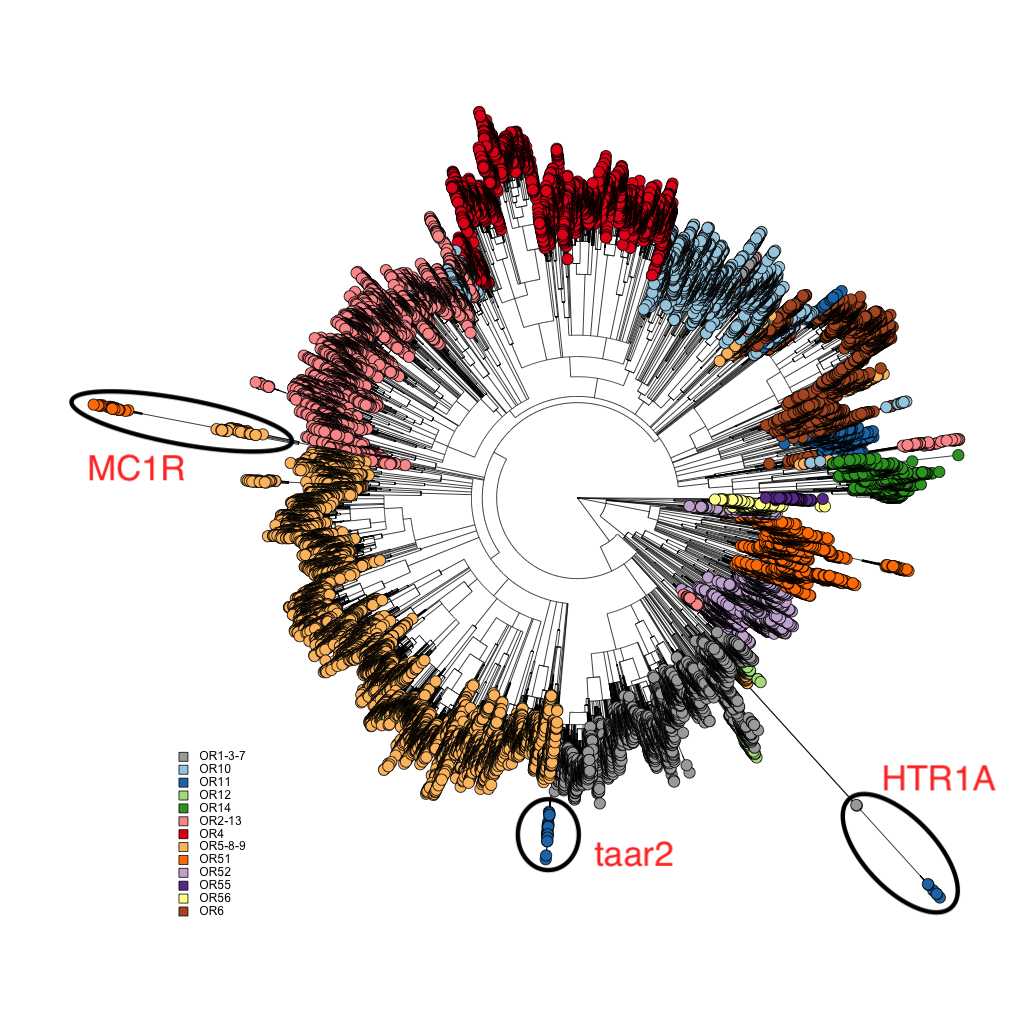

Supplement: evad197_Supplementary_Data [file evad197_supplementary_data.zip › S2_RodentOR_gene_tree.png]

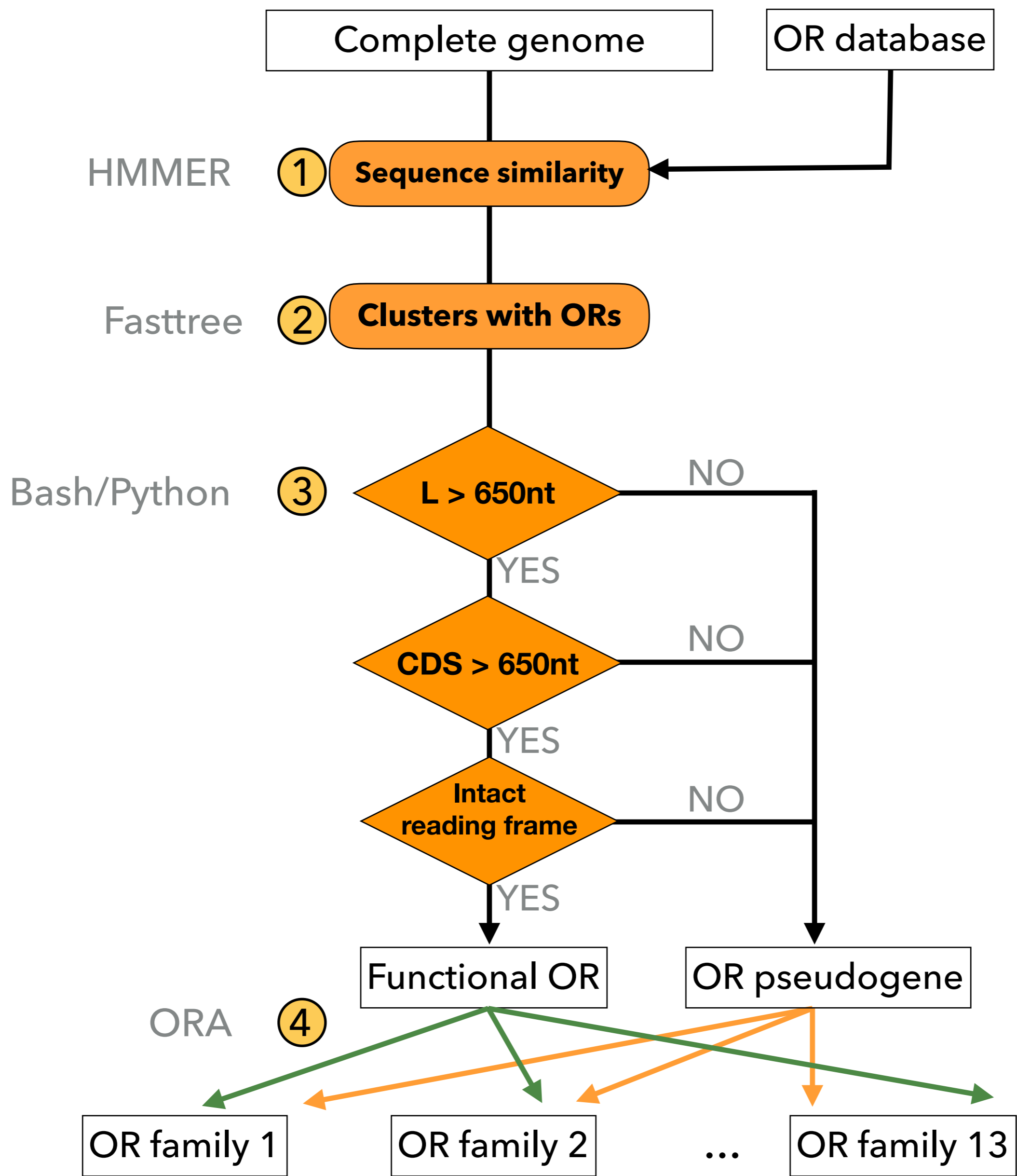

Supplement: evad197_Supplementary_Data [file evad197_supplementary_data.zip › S3_Pipeline.pdf]
